# Superficial Cervical Plexus Block in Selective Cases of Oral and Maxillofacial Surgery as an Alternative to General Anesthesia: A Case Presentation

**DOI:** 10.7759/cureus.21371

**Published:** 2022-01-18

**Authors:** Ravi Raja K Saripalli, Srikanth Kasaraneni, Sai S Yadavilli, Leela Subhashini C Alluri

**Affiliations:** 1 Department of Oral and Maxillofacial Surgery, St. Joseph Dental College, Eluru, IND; 2 Department of Periodontics, Meharry Medical College School of Dentistry, Nashville, USA

**Keywords:** superficial cervical plexus anatomy, local anesthesia, mandibular fracture, superficial cervical plexus block, regional anesthesia

## Abstract

The superficial cervical plexus block (SCPB) is well acknowledged, accepted for regional anesthetic for the submandibular area, neck region, and ear lobe. It is a possible valuable anesthetic technique in individuals to be operated on with conditions such as ear lobe lacerations, submandibular abscesses, and trauma to the mandibular or the neck region. Its application in oral and maxillofacial surgery is for various surgical procedures in the peri-mandibular area, excision of superficial lesions in the mandibular, ear lobe, neck region, and suturing of the skin in the corresponding region. We illustrate a case of non-union of mandibular fracture operated under superficial cervical plexus block without any complications.

## Introduction

The superficial cervical plexus block (SCPB) is a simple technique, easy to perform, and used in preference to general anesthesia (GA) in selected cases; however, it is often left unnoticed [1]. GA is used in modern-day medicine as a simple and relatively safe method to achieve surgical anesthesia. However, GA has many drawbacks: high cost, the requirement of several well-trained crew and higher-end equipment, morbidity, and mortality. Stress-free anesthesia can be provided using regional anesthesia as its simple technique, lower catecholamine release, decreased blood loss due to local vasoconstrictors and sympathetic blockade, and negligible morbidity rates with appropriate dosages of the local anesthetic (LA) [2]. Halstead, in 1884 was the first to present and perform the cervical plexus block. Later the posterior route was described by Kappis in Germany [3]. Heidenhein launched the lateral approach technique, and Labat popularized it in the USA [4]. The SCPB technique is well elicited for regional anesthesia of the perimandibular region, the neck, and the ear lobe. It is a potentially valuable anesthetic technique for patients to be operated on with conditions such as ear lobe lacerations, submandibular abscesses, and injuries to the mandibular region or the neck [5]. Its use in oral and maxillofacial surgery has been for various surgical procedures in the perimandibular area, excision of superficial lesions in the mandible, ear lobe, or neck region, and suturing of the skin in the corresponding region [6]. Minor oral and maxillofacial surgical procedures are commonly executed in an ambulatory care setting. Regional anesthesia is incomparably the best-established procedure to sedate patients in advanced office-based procedures. Anesthesia of the teeth, adjacent soft and hard tissues of both the maxilla and mandible can be obtained by employing numerous techniques. The procedure and the location determine the anesthetic technique [6].

Anatomy and landmarks of the cervical plexus

The cervical plexus (CP) is a network of nerve fibers formed by the union of the upper four cervical nerves of the anterior division. CP is located on the anterior surface of the upper four cervical vertebrae and underneath the sternocleidomastoid (SCM) muscle. Furthermore, it lies over the levator anguli scapulae and scalenus medius muscles and arises from the posterior border of the SCM. The spinal nerve is formed by the union of its dorsal and ventral roots that pass through the intervertebral foramen. The anterior rami of C2 through C4 forms the CP (with C1 being a motor root component and is not anesthetized by SCPB). The superficial branches are superficial cervical plexus (SCP), and deep cervical plexus (DCP) are dispensed from CP. The SCP branches emerge out from the posterior border of the sternocleidomastoid muscle into four nerves to innervate the superficial structures of the neck, head, shoulder region, and corresponding skin in the region. The four sensory nerve branches of the SCP are the lesser occipital, greater auricular, transverse cervical, and supraclavicular nerves. Originating from the C2, the lesser occipital nerve climbs up to the sternocleidomastoid’s posterior border to innervate the area behind the ear, superior-inferiorly like a band. The greater auricular nerve and transverse cervical nerve arise from C2 and C3. The greater auricular supplies sensation to the superficial structures on the parotid gland region posteroinferior from the ear surface to the angle of the mandibular region. The transverse cervical nerve, also called the anterior cutaneous nerve, splits into two branches-anterior and posterior after penetrating the platysma muscle. It provides sensation from the sternal region to the angle of the mandibular region. The supraclavicular nerve branches emerge from the C3 and C-4, perforating the platysma muscle and supplying inferiorly to the clavicular region, the second rib region, and superolateral the deltoid muscle. A local anesthetic agent can easily block all these nerves that make up the SCP. The branches that arise from DCP innervate deeper structures of the neck, the diaphragm, and muscles in the anterior neck region. The anterior cutaneous and supraclavicular nerve supply sensation directly to the trapezius or dispense a branch to the XI cranial nerve [6].

## Case presentation

A fifty-four-year-old male patient reported to the department of oral and maxillofacial surgery with pain and mobility of the jaw. The patient has a history of getting operated on in a private hospital for a lower jaw fracture under general anesthesia six months ago due to a road traffic accident. The patient gives no significant past medical history. Upon examination, no gross asymmetry of the face was seen, skin and soft tissue had no abnormalities. No clicking or crepitus while opening or closing mouth in the temporomandibular joints (TMJ). The mouth opening was 40 mm. There was no derangement of occlusion; however, there was tenderness on palpation in the right angle region of the mandible with grade II mobility in relation to tooth number 31. Bimanual palpation revealed segmental mobility of the lower jaw at tooth number 31 region.

Investigations

**Figure 1:**
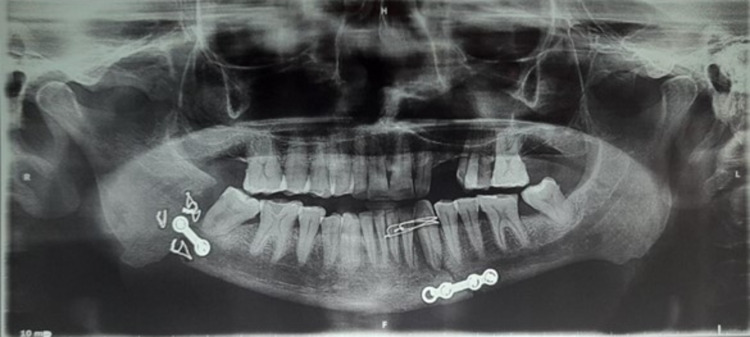
Pre-operative orthopantomogram (OPG) showing the discontinuity of the bony fragments at the right angle region of the mandible

The orthopantomogram (OPG) revealed the presence of multiple transosseous wires, a two-hole plate at the right angle region, and a four-hole plate at the left parasymphysis of the mandible. Additionally, interdental wiring involved tooth number 22 to tooth number 24. A wide discontinuity of the bony fragments was still appreciable at the right angle region of the mandible (Figure 1). 

Treatment

**Figure 2:**
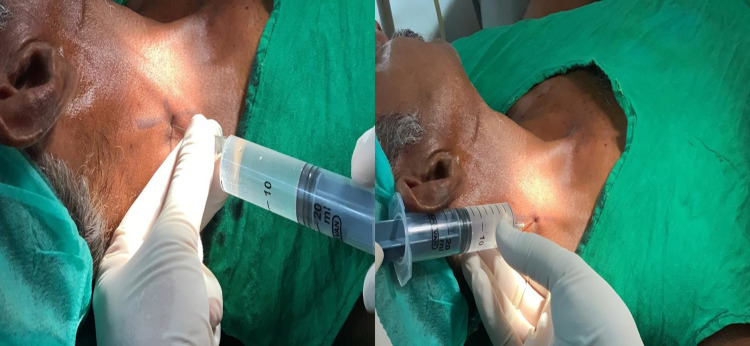
Administration of the superficial cervical plexus block (SCPB)

A diagnosis of non-union of the right angle of the mandible was made based on the clinical and radiological findings. The patient was informed to be operated on for non-union of angle fracture under local and regional anesthesia, and consent was obtained. Under strict aseptic conditions and stable vitals monitoring, the patient was administered with the SCPB (Figure 2)

The technique of the superficial cervical plexus block (SCPB)

**Figure 3:**
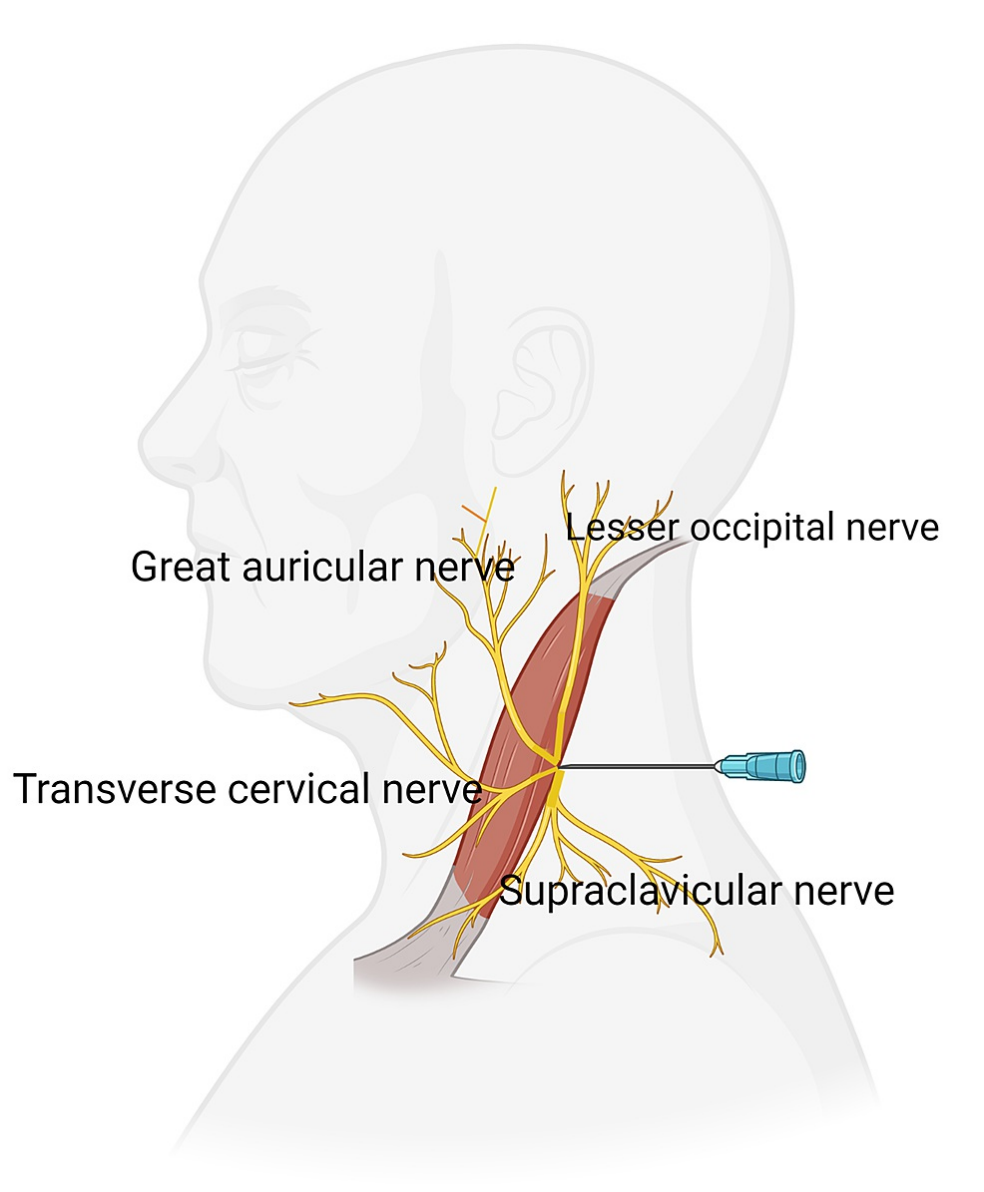
Technique of the superficial cervical plexus block The figure is created with BioRender^©^

The patient was made to lie in a supine position with a towel under his head. The patient’s head was turned slightly toward the non-anesthetized side. In counter to gentle resistance from the hand of the anesthetist, the patient was instructed to lift the head. A Valsalva maneuver was performed simultaneously to trace the outline of the sternocleidomastoid muscle and find the location of the external jugular vein. The midpoint of the posterior border of the sternocleidomastoid was located and marked the external jugular vein that crosses the muscle border. A 22-gauge needle of around 4 cm length was advanced into the sub fascia 2-3 cm superiorly and inferiorly along the border of the sternocleidomastoid muscle with infiltration of 5-10 ml of local anesthetic. Determination of adequacy of the block was done after 10-15 minutes after injecting the local anesthetic (Figure 3) [2].

**Figure 4:**
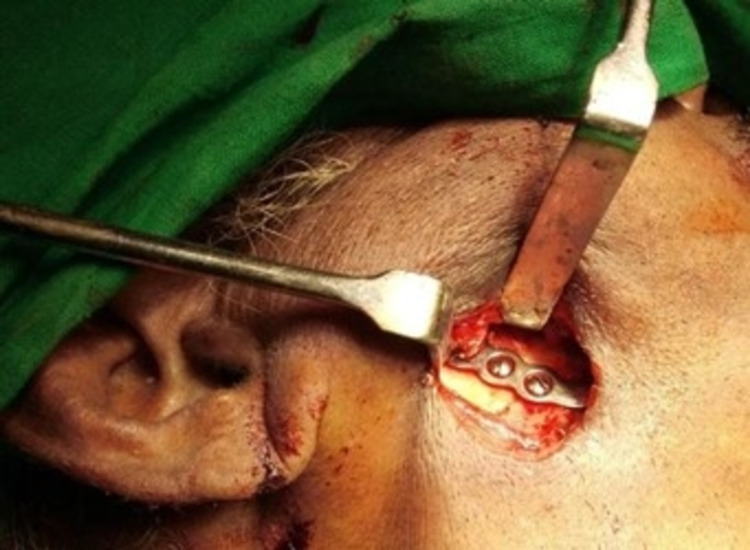
Operating under superficial cervical plexus block (SCPB)

**Figure 5:**
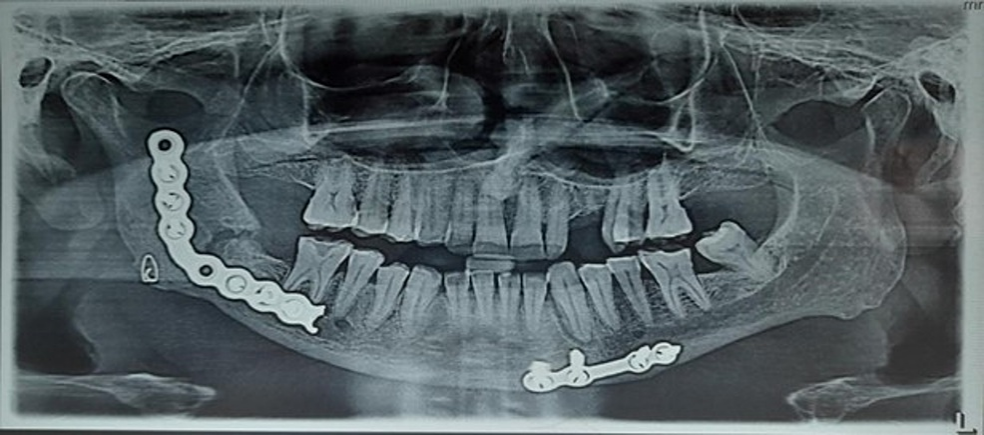
Post-operative orthopantomogram (OPG)

Additionally, the mandibular nerve block was performed using a mixture of 3:1 ratio of 0.5% bupivacaine and 2% lignocaine. The onset of action of the SCPB was within five minutes, and the mandibular nerve block was within three minutes. Using the pre-existing scar, an extraoral incision was made to explore the right angle of the mandible. The pre-existing transosseous wiring and two-hole plate removal were done, retaining a single transosseous wire at the angle of the mandible. After curettage and anatomical approximation of the fragmented ends, a reconstruction plate (2.5mm) was placed to achieve fixation (Figures 4, 5).

.

Besides, the old plate at the left parasymphysis region was removed and replaced with a new plate with proper fixation. Pain during the procedure was measured using the Visual Analog Scale (VAS), which the patient elicited as a two.

Outcome and follow-up:

The post-operative healing was satisfactory. The SCPB lasted for six hours, acting as post-operative analgesia. The vitals during and after the procedure were stable. The patient was reviewed for 18 months every three months and had no post-operative complications.

## Discussion

The SCPB produces adequate regional anesthesia to the neck and maxillofacial region. Aiming the superficial branches of the CP at the mid-point of the sternocleidomastoid's posterior border, the SCPB is described as a subcutaneous injection conventionally. The SCPB can be executed with or without an ultrasound guide using the conventional subcutaneous injection technique. Using suitable landmarks, one or more branches of the SCPB can be blocked depending on the procedure performed in the neck and head region. Unlike the deep cervical plexus block (DCPB), the SCPB is easy to master [7]. While performing an SCPB, to avoid the anesthesia of the deep cervical plexus and its side effects, it is essential to place the needle tip in the subcutaneous tissue.

The other aspect of SCPB is analgesia. SCPBs, when performed unilaterally or bilaterally, can provide excellent postoperative analgesia after a variety of head and neck surgical procedures such as parathyroidectomy, surgeries of the tympanomastoid region, thyroidectomy, discectomy in the anterior cervical region and fusion, occipital and infratentorial craniotomy. External ear surgery, such as ear lobe lacerations or otoplasty, can be used solitarily as an anesthetic modality. By specifically blocking the supraclavicular nerve, SCPBs can also be used concomitantly with other blocks for surgeries in the clavicular, shoulder, upper chest wall, and breast regions [8]. An essential component in the oral and maxillofacial surgery branch is pain management. Conventional local anesthetic blocks delivered properly can offer sufficient anesthesia. However, it does not provide requisite analgesia in certain circumstances, such as perimandibular space infections where abscesses invade the deeper facial planes. These perimandibular and cervical lesions essentially need prosection in the deeper tissue planes and trauma of the body, and angle of the mandibular region. In these conditions, GA is usually preferred, although its disadvantages are high cost, morbidity, and chances of mortality. SCPB technique can ensure safety and patient comfort to execute surgical procedures in the neck and perimandibular area [2].

SCPB provides excellent analgesia during skin incisions and tissue prosection. Combination SCPB with other traditional nerve blocks in the mandibular region provided good anesthesia with successful results, as seen by Kanthan [2]. Furthermore, Arun et al. [9] used this block for incision & drainage of Ludwig's angina. They concluded that even with a limited setup of resources, SCPB permits surgical decompression for cases requiring incision and drainage. Koshy et al. [10] used SCPB for tracheostomy and concluded that SCPB makes the patient more cooperative and comfortable during the procedure. Additionally, they stated that a combination of SCPB with transtracheal installation for semi-emergency tracheostomies provided safe, easy, swift, and eminent analgesia. Thorough knowledge of the regional anatomy, landmarks, and the application of the right technique for SCPB is vital to achieving good clinical results. Several probable adverse effects and complications such as LA toxicity, hematoma, infection, transient phrenic nerve palsy, and nerve injury are known with SCPB, although if properly managed, they are commonly rare [11].

No unfavorable incidents were recorded in the above-stated case. The primary complications of the SCPB arise only with an unintentional deeper deposition of local anesthetic leads to anesthesia of the deeper structures, which includes the brachial plexus, phrenic nerve, and the recurrent laryngeal nerve. Still, these situations are infrequent and can easily be kept away through proper technique and following the block precautions [12].

## Conclusions

The SCPB is a simple and easy technique that can be widely used following proper anatomical landmarks and techniques. It offers adequate regional anesthesia in the maxillofacial region. When concomitantly used with other mandibular nerve blocks, SCPB has a high success rate, low complications, higher acceptance of patients, and negligible chances of morbidity and mortality, which is why it can be used to replace GA in selected cases of maxillofacial surgery. It can be used not only for surgical anesthesia but also for various other maxillofacial procedures requiring postoperative analgesia.
